# Nanopharmacology as a new approach to treat neuroinflammatory disorders

**DOI:** 10.1515/tnsci-2022-0328

**Published:** 2023-12-16

**Authors:** Sebastián García Menéndez, Walter Manucha

**Affiliations:** Instituto de Medicina y Biología Experimental de Cuyo (IMBECU), Consejo Nacional de Investigaciones Científicas y Tecnológicas (CONICET), Mendoza, Argentina; Área de Farmacología. Departamento de Patología, Facultad de Ciencias Médicas, Universidad Nacional de Cuyo, Mendoza, Argentina

**Keywords:** neuroinflammation, neurodegeneration, nanopharmacology, nanoparticles, drug delivery, Alzheimer’s disease, Parkinson’s disease, multiple sclerosis, schizophrenia, bipolar disorder

## Abstract

Neuroinflammation, a complex process involving the activation of microglia, astrocytes, and other immune cells in the brain, plays a role in neurodegeneration and psychiatric disorders. Current therapeutic strategies for neuroinflammation are limited, necessitating the development of improved approaches. Nanopharmacology offers unprecedented opportunities to access and treat neuroinflammatory disorders at the brain level. Nanoscaffolds can target specific cells or tissues and protect drugs from degradation or elimination, making them ideal candidates for treating neurodegenerative and psychiatric diseases. Recent advancements in nanoparticle development have enabled the targeting of microglia, astrocytes, and other immune cells in the brain, reducing neuroinflammation and protecting neurons from injury. Nanoparticles targeting specific neurons have also been developed. Clinical trials are in progress to evaluate the safety and efficacy of nano drugs for treating neuroinflammatory, neurodegenerative, and psychiatric diseases. The successful development of these nanodrugs holds immense promise for treating these devastating and increasingly prevalent conditions. On the other hand, several limitations and unanswered questions remain. First, the long-term effects of nanoparticles on the brain need to be thoroughly investigated to ensure their safety. Second, optimizing the targeting and delivery of nanoparticles to specific brain regions remains a challenge. Understanding the complex interplay between nanoparticles and the brain’s immune system is crucial for developing effective nanotherapies. Despite these limitations, nanopharmacology presents a transformative approach to treating neuroinflammatory disorders. Future research should address the aforementioned limitations and further elucidate the mechanisms of nanoparticle-mediated therapy. The successful development of safe and effective nanodrugs can revolutionize the treatment of neuroinflammatory disorders, alleviating the suffering of millions.

## 1 Introduction

Neuroinflammatory, neurodegenerative, and psychiatric disorders pose a significant global health challenge, affecting millions worldwide. These debilitating conditions are characterized by the progressive loss of neurons and declining cognitive function, motor control, and behavior. Current treatment options are often ineffective or associated with severe side effects. For instance, anti-inflammatory drugs can induce gastrointestinal distress, while neuroprotectants may exhibit neurotoxicity [1].

In recent years, nanopharmacology has emerged as a promising therapeutic approach for these challenging diseases. Nanoparticles, with their unique size-dependent properties, offer the potential to deliver drugs directly to specific cells or tissues in the brain, enhancing efficacy and minimizing side effects. This targeted delivery strategy holds particular promise for neuroinflammatory, neurodegenerative, and psychiatric diseases due to the challenges of conventional drug delivery across the blood–brain barrier (BBB) [1,2].

Several advantages make nanoparticles well-suited for treating these neurological disorders. First, they can improve the solubility and stability of drugs, particularly those with poor pharmacokinetic properties. Second, nanoparticles can be functionalized to target specific cells or tissues in the brain, ensuring precise drug delivery to the desired site of action. Third, nanoparticles can effectively cross the BBB, overcoming a significant hurdle in conventional drug delivery to the central nervous system (CNS) [3].

**Figure 1:**
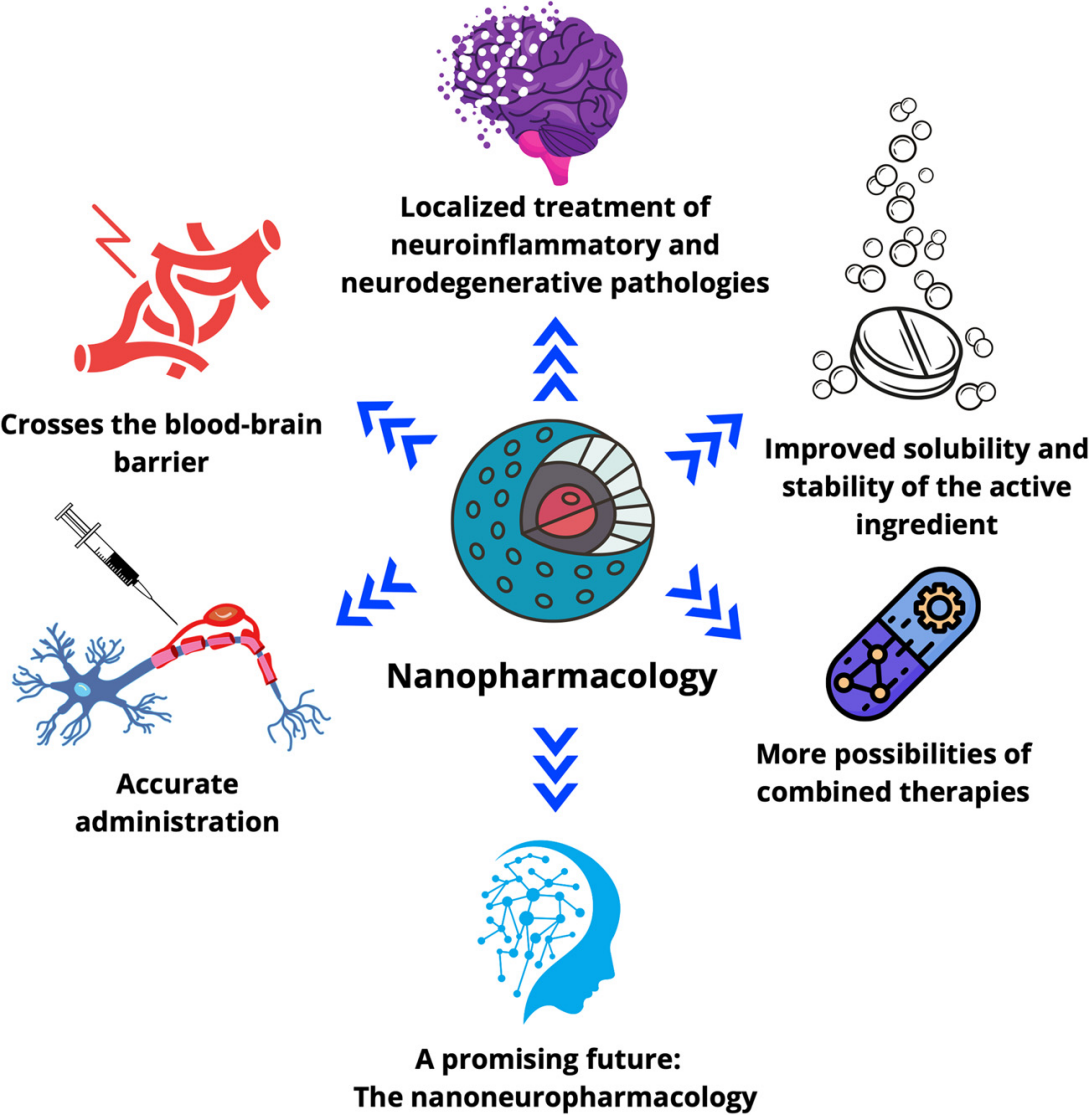
This graphical overview presents the key benefits of nanoparticle drug delivery for neuroinflammatory and neurodegenerative diseases. From improved drug solubility and stability to precise administration in the nervous system and overcoming the BBB, this visual schematic highlights how nanoparticles are revolutionizing the field of nanopharmacology. Additionally, it illustrates the potential of nanoparticles for combination therapies and provides a promising glimpse into the future of these innovative treatments.

Significant advancements have been made in developing nanoparticulate drug delivery systems for treating neuroinflammatory, neurodegenerative, and psychiatric diseases. Nanoparticles have been successfully employed to deliver anti-inflammatory drugs to the brain for the treatment of multiple sclerosis (MS) and Alzheimer’s disease (AD) [4]. Similarly, nanoparticles have been utilized to provide neuroprotective agents to the brain in treating Parkinson’s disease and stroke [5] (Figure 1).

In this mini-review, we explore the therapeutic potential of nanopharmacology for neuroinflammatory, neurodegenerative, and psychiatric diseases. We delve into the advantages of nanoparticulate drug delivery systems for these conditions and address the challenges that must be surmounted before nanopharmacology can be widely adopted in clinical practice. We highlight the novelty of many works in developing novel nanoparticulate drug formulations for treating these complex neurological disorders. Conversely, we acknowledge several limitations and unanswered questions that warrant further mechanistic and clinical investigations.

### 1.1 Advantages of nanoparticle drug delivery for neuroinflammatory and neurodegenerative diseases?

Neuroinflammatory and neurodegenerative diseases represent a significant public health burden, characterized by progressive loss of neurons and decline in cognitive function, motor control, and behavior [6]. While traditional treatments are somewhat effective, they generally only slow the progression of the disease and do not fully reverse the underlying damage.

In this challenging landscape, nanopharmacology is emerging as a promising strategy. Nanoparticles offer numerous advantages in drug delivery for neuroinflammatory and neurodegenerative diseases. First, they improve the solubility and stability of drugs, which is essential for drugs with low solubility or stability, protecting them from degradation and improving their therapeutic efficacy [7,8]. Second, nanoparticles allow for precise drug delivery to specific cells or tissues, such as the CNS. By modifying them with ligands or antibodies, nanoparticles can bind to receptors or markers on the cell surface, minimizing off-target effects [8,9]. Third, nanoparticles overcome the challenge of the BBB, a significant obstacle to drug delivery to the CNS. Designed nanoparticles can cross or disrupt the BBB, facilitating efficient drug delivery. Strategies such as passive targeting, active targeting, and BBB disruption help transport nanoparticles across the BBB [10].

In addition, nanoparticles ensure controlled and sustained drug release, prolonging therapeutic effects and reducing the frequency of administration, which is essential for chronic neuroinflammatory and neurodegenerative diseases [8]. Additionally, nanoparticles have the potential to carry multiple drugs or combination therapies, promoting synergistic effects and better outcomes, which is especially beneficial for complex diseases that involve various processes or targets [11].

Understanding the molecular and cellular mechanisms underlying the interplay between neuroinflammation and neurodegeneration is crucial for developing new therapies to prevent and treat pathologies arising from these two pathological conditions. In this sense, some nanotech-based treatments have also been developed to modulate the neuroinflammatory response and protect neurons from cellular damage [12].

In summary, nanoparticle-based drug delivery (NDD) for neuroinflammatory and neurodegenerative diseases offers benefits such as improved drug solubility and stability, precise administration, BBB penetration, sustained release, and potential for combination therapies. This approach shows promise for effectively addressing these challenging conditions [13].

## 2 Nanopharmacology for the treatment of neuroinflammation

Neuroinflammation is a critical factor in the development of many neurodegenerative diseases. The activation of microglia, astrocytes, and other immune cells in the brain characterizes it. These activated cells release inflammatory mediators that can damage neurons and promote neurodegeneration [6]. Thus, the role of neuroinflammation in neurodegenerative diseases is a complex and multifaceted issue, and further research is needed to understand the mechanisms involved fully. However, neuroinflammation is a critical factor in the progression of these diseases, and targeting neuroinflammation may hold promise for developing new therapies. In this sense, nanoparticles can deliver drugs targeting and reducing neuroinflammation. For example, nanoparticles have been developed to provide the brain with anti-inflammatory medications, such as ibuprofen and dexamethasone. These nanoparticles effectively reduce neuroinflammation in animal models of AD, Parkinson’s disease, and MS [14].

Recently, through a combination of nanotechnology and an innovative nano vector, the leukocyte chemotactic peptide (fMLP), the impact of C16 peptide and angiopoietin 1 in MS models were evaluated. The results indicate that these compounds managed to attenuate neuronal inflammation, axonal demyelination, neuronal apoptosis, and vascular leakage, in addition to preserving the integrity of the BBB. The use of the fMLP nano vector proved particularly effective compared to traditional formulations, highlighting its therapeutic potential in MS and offering a promising path for future treatment strategies [15].

In contrast, pathologies with a more acute course, such as intracerebral hemorrhage (ICH), continue to be a significant challenge due to surgical risks and mainly to the difficulty of removing the deep hematoma. A promising approach that seems to overcome this drawback is the activation of peroxisome proliferator-activated receptor gamma (PPARγ). A recent study presents a magnetic nanocarrier with a PPARγ agonist (15d-PGJ2-MNPs) that targets the hematoma area after intravenous injection. Focused ultrasound (FUS) application enhances drug diffusion, activating PPARγ in macrophages near the hematoma for better clearance. This approach showed benefits in terms of reduced brain damage, neuroinflammation, and improvement of sensory and motor functions in a mouse model of ICH. This method combines magnetic targeting with FUS and shows considerable potential for treating cerebral hemorrhages [16]. In ischemic stroke, oxidative stress and mitochondrial damage are vital issues. One study used cerium oxide nanoparticles to carry dl-3-*n*-butylphthalide (NBP-CeO_2_ NPs) and treat this problem. NBP-CeO_2_ NPs scavenged free radicals in brain cells and neurons, preserving mitochondrial function and the integrity of the BBB. In a mouse stroke model, these nanoparticles protected mitochondria and reduced brain injury, edema, and inflammation. Furthermore, they improved long-term brain function by promoting angiogenesis. These nanoparticles offer a promising therapy for stroke by combining antioxidants and neurovascular repair [17].

Finally, different clinical trials are underway to test the safety and efficacy of nanodrugs in treating neuroinflammatory pathologies. One of these articles examined the effects of omega-3 fatty acids and nano-curcumin, known for their anti-inflammatory and neuroprotective properties, in migraine sufferers. They showed that combining omega-3 fatty acids and nano-curcumin reduced the expression of inflammatory genes, such as TNF-α and COX-2/iNOS, and their levels in the blood. Furthermore, this combination significantly decreased the frequency of migraine attacks, severity, and duration [18,19,20].

## 3 Nanopharmacology for the treatment of neurodegeneration

Nanoparticles can also be used to deliver drugs that can protect neurons from damage and promote their survival. For example, nanoparticles have been developed to provide the brain with antioxidants, such as vitamin E and glutathione. These antioxidants have been shown to protect neurons from damage caused by free radicals, which are unstable molecules that can damage DNA and proteins [21,22]. In the same way, nanoparticles have also been developed to deliver drugs that can promote the growth and repair of neurons, and more specifically, nanoparticles have been designed to provide growth factors, such as brain-derived neurotrophic factor (BDNF), to the brain [23]. This development could help prevent or reverse brain damage caused by diseases such as AD, Parkinson’s disease, and stroke. Nanoparticles could also be used to deliver other drugs to the brain, such as anti-inflammatory or neuroprotective drugs.

In parallel and close relation, mitochondrial dysfunction has been the subject of multiple investigations to understand the intimate mechanisms of inflammatory and oxidative diseases typical of aging. Mitochondrial dysfunction is also a key feature in AD but current treatments have limitations. In a recent study, extracellular vesicles derived from modified mesenchymal stem cells were used to deliver an enzyme (SHP2) to the brain of AD mice. This induced the clearance of damaged mitochondria in brain cells, reducing apoptosis and inflammation. This improved synapse loss and cognitive function in AD mice, showing a promising approach to treating the disease [24]. In contrast, another work also showed decreased oxidative markers and improved mitochondrial function in these AD models, using a treatment of formulated resveratrol and selenium nanoparticles (RSV-SeNPs). They found that these nanoparticles also reduce the accumulation of amyloid beta protein and regulate signaling pathways that contribute to the disease by decreasing brain inflammation. They also promote neurite outgrowth and improve cognitive function. RSV-SeNPs show therapeutic potential for this disease by addressing multiple aspects of pathology [25]. Another compound that is becoming increasingly relevant is engeletin due to its antioxidant and anti-inflammatory properties that act through the Keap1/nrf2 pathway. However, its solubility and permeability limit its delivery to the brain. A study has proposed a theoretical formulation of engeletin lipid nanocarriers for treating Huntington’s disease. These nanostructures could facilitate the delivery of the active principle to the brain, thanks to using a composition similar to the natural lipids of the brain, increasing its bioavailability [26]. In this vein, NDD technology has shown promise for treating inflammatory depression by crossing the BBB. In parallel, it was experimented with melanin-like nanoparticles, called polydopamine (PDA NPs) (∼250 nm), which can cross the BBB. These PDA NPs contain abundant phenolic hydroxyl groups that function as excellent free radical scavengers to attenuate cell damage caused by reactive oxygen species or acute inflammation. The experiments showed that PDA NPs reverse depressive behavior and reduce peripheral and central inflammation in a mouse model. They also have good biocompatibility. Although its potential as a treatment for inflammatory depression is envisioned, more research is required for its clinical use [27]. Finally, recent findings on nanomedical strategies and applications for the encapsulation of cannabinoids have become the objective of improving their therapeutic use and their application in addiction therapy. Nanoformulations enhance the pharmacokinetics and pharmacodynamics of cannabinoids, offering greater efficacy, lower toxicity, and more controlled/prolonged release than cannabinoids in free form [9].

## 4 Discussion

NDD is a promising new approach for treating neuroinflammatory and neurodegenerative diseases. This new technology offers several advantages over traditional drug delivery methods, including improved solubility and stability. Nanoparticles can enhance the solubility and stability of drugs, which is essential for drugs with low solubility or stability. This can improve the therapeutic efficacy of drugs and reduce the risk of side effects. In addition, nanoparticles can target specific cells or tissues, such as the brain [28]. This can help to reduce off-target effects and improve the efficacy of drug delivery with the ability to overcome the BBB. Nanoparticles can be designed to cross the BBB, which can facilitate drug delivery to the brain. Parallelly, nanoparticles can be designed to release drugs in a controlled and sustained manner. This can help to improve the therapeutic efficacy of drugs and reduce the frequency of administration. Nanoparticles can deliver multiple drugs or combination therapies, promoting synergistic effects and improving outcomes [29].

NDD has shown promise in preclinical models of various neuroinflammatory and neurodegenerative diseases, including AD, Parkinson’s disease, MS, and stroke. Clinical trials are in progress to evaluate the safety and efficacy of NDD in treating these diseases. Despite the promise of NDD, several challenges still need to be addressed, including biocompatibility and side effects. Nanoparticles must be biocompatible and have minimal side effects. This critical challenge must be addressed before NDD can be used in clinical practice. Nanoparticles must effectively reach the target site in the brain [30]. This is a complex challenge that requires further research. Nanoparticles must be designed to efficiently load and release drugs in a controlled and sustained manner. This is a challenging engineering problem that needs to be addressed.

Despite these challenges, NDD is a promising new approach for treating neuroinflammatory and neurodegenerative diseases. As research progresses, NDD will likely play an increasingly important role in treating these diseases [31].

In addition to the challenges mentioned above, NDD also faces the challenge of treating complex diseases that involve multiple pathological processes. Nanoparticles have the potential to address these complexities by delivering multiple drugs or targeting multiple pathways simultaneously [32]. However, this is a complex challenge that requires further research. The ability of nanoparticles to modulate neuroinflammation and protect neurons further enhances their therapeutic potential. Nanoparticles can be designed to deliver drugs that reduce inflammation, protect neurons from damage, or promote neuron growth and repair [33]. These properties have the potential to slow or even reverse the progression of neuroinflammatory and neurodegenerative diseases. As research progresses, NDD is likely to play an increasingly important role in the treatment of neuroinflammatory and neurodegenerative diseases, allowing the provision of new and effective therapies for these devastating diseases [34].

## 5 Conclusion

In nanopharmacology, nanoparticles are emerging as a promising platform for treating neuroinflammatory and neurodegenerative diseases, representing a significant challenge for public health due to the loss of neurons and decreased brain function. These tiny structures address these challenges in multifaceted ways.

First, they improve the solubility and stability of drugs, overcoming the limitations of conventional therapies. In addition, they allow precise delivery of drugs to specific cells or tissues in the CNS, minimizing unwanted side effects. They overcome a crucial BBB that hinders efficient drug delivery to the CNS using passive/active targeting and BBB disruption.

Additionally, nanoparticles ensure a controlled and sustained release of drugs, which reduces the frequency of administration, vital for chronic diseases. Finally, its ability to transport multiple drugs or combined therapies promotes synergistic effects, especially beneficial for complex conditions.

**Figure 2:**
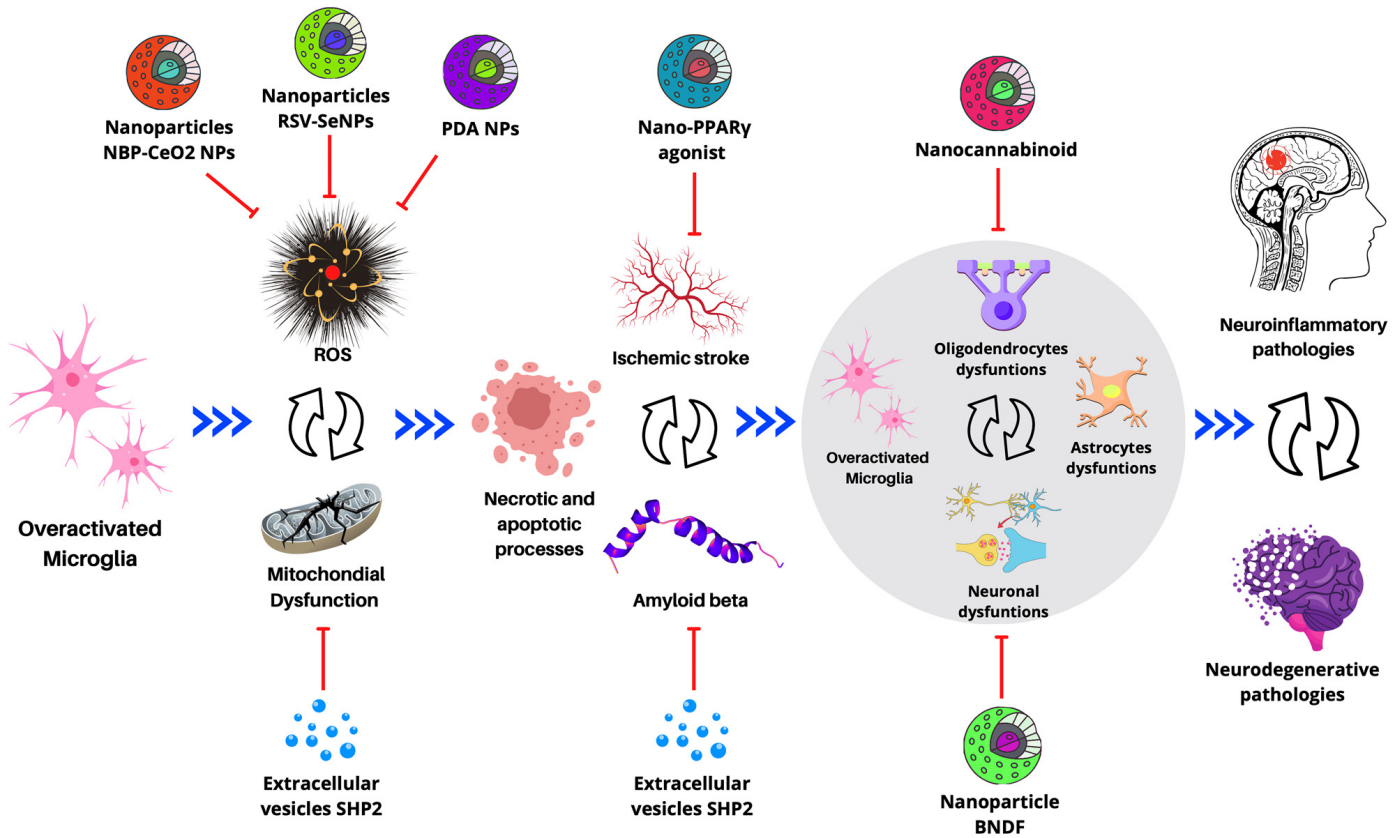
Diagram of the progression of neuroinflammatory and neurodegenerative processes originating from microglial overactivation. The nanodrugs under investigation are emphasized, aiming to halt or reverse these pathological processes. Blue arrows: activation; red lines: inhibition; double arrows: feedback. PPARγ, peroxisome proliferator-activated receptor gamma; NBP-CeO_2_ NPs, cerium oxide nanoparticles to carry dl-3-*n*-butylphthalide; BDNF, extracellular vesicles deliver an enzyme (SHP2); RSV-SeNPs, resveratrol and selenium nanoparticles; PDA NPs, melanin-like nanoparticles called polydopamine.

These advances are revolutionizing how we deal with diseases like Alzheimer’s, MS, and ICH. As research and refinement of these technologies continue, it is plausible to expect increasingly effective and personalized therapies, which could significantly improve the quality of life for patients facing these challenging conditions (Figure 2).

Despite the promising potential of nanotechnology in treating neuroinflammatory diseases, several limitations must be addressed. While nanoparticles offer a targeted approach to drug delivery, their interactions with biological systems still need to be fully understood. Long-term exposure to nanoparticles could lead to unintended toxicity or other adverse effects. Nanoparticles can improve drug delivery across the BBB but achieving consistent and controlled penetration remains a challenge. The complex structure of the BBB and the diversity of nanoparticle properties can hinder effective delivery to specific brain regions. Also, some nanoparticle formulations can cross the BBB but efficiency and specificity still need to be improved. Nanoparticles may activate the immune system, leading to inflammation or other adverse reactions. Careful design and surface modifications are crucial to minimize immune responses and ensure biocompatibility. Translating nanotechnological developments from the lab to clinical practice requires extensive testing and rigorous regulatory approval processes. Demonstrating safety, efficacy, and long-term effects in human trials is essential for widespread adoption [35].

## 6 Future perspectives

Future research directions include developing nanoparticles with tailored properties, such as size, shape, surface modifications, and drug loading, to optimize efficacy and minimize side effects. Also, utilizing active targeting mechanisms, such as ligand–receptor interactions or magnetic guidance, to direct nanoparticles to specific brain regions with greater precision. Combining nanoparticles with other therapeutic modalities, such as gene therapy or immunotherapy, enhances treatment outcomes and addresses complex disease mechanisms. Developing biodegradable and biocompatible nanoparticle formulations to minimize long-term toxicity and improve safety profiles. Utilizing advanced imaging techniques to monitor nanoparticle distribution, biodegradation, and therapeutic effects *in vivo*. Evolving personalized medicine approaches based on patient-specific factors, such as genetic background and disease progression, to optimize treatment efficacy and minimize adverse reactions [36].

By addressing the current limitations and pursuing these promising research directions, nanotechnology has the potential to revolutionize the treatment of neuroinflammatory diseases and improve the quality of life for patients worldwide.
